# Influence of the Elastoplastic Strain on Fatigue Durability Determined with the Use of the Spectral Method

**DOI:** 10.3390/ma13020423

**Published:** 2020-01-16

**Authors:** Michał Böhm, Mateusz Kowalski, Adam Niesłony

**Affiliations:** Department of Mechanics and Machine Design, Faculty of Mechanical Engineering, Opole University of Technology, Ulica Prószkowska 76, 45-758 Opole, Poland; m.kowalski@po.opole.pl (M.K.); a.nieslony@po.opole.pl (A.N.)

**Keywords:** spectral method, fatigue of materials, steel 0H18N9, frequency domain

## Abstract

The paper presents experimental static and fatigue tests results under random loading conditions for the bending of 0H18N9 steel. The experimental results were used in performing calculations, according to the theoretical assumptions of the spectral method of fatigue life assessment, including elastoplastic deformations. The presented solution extends the use of the spectral method for material fatigue life assessment, in terms of loading conditions, above Hooke’s law theorem. The work includes computational verification of the proposal to extend the applicability of the spectral method of determining fatigue life for the range of elastoplastic deformations. One of the aims of the proposed modification was to supplement the stress amplitudes used to calculate the probability density function of the power spectral density of the signal with correction, due to the plastic deformation and its use for notched elements. The authors have tested the method using four of the most popular probability density functions used in commercial software. The obtained results of comparisons between the experimental and calculation results show that the proposed algorithm, tested using the Dirlik, Benasciutti–Tovo, Lalanne, and Zhao–Baker models, does not overestimate fatigue life, which means that the calculations are on the safe side. The obtained results prove that the elastoplastic deformations can be applied within the frequency domain for fatigue life calculations.

## 1. Introduction

The determination of the fatigue life of construction materials and working machines is currently a constantly evolving area of interest for many scientists. A particularly interesting case is the determination of durability under random loads. The use of an appropriate method, in combination with the finite element method (FEM), often allows us to visualize durability results in the form of colored strength maps. The literature of the subject presents two methods for determining fatigue life under operational loads—the cycle counting method [1,2], in which calculations are carried out in the time domain, and the spectral method [3,4], which is determined in the frequency domain. The basic difference between these methods is the way in which the distribution of amplitudes from the load course is determined, which, in a general case, may manifest a random character [5]. In the cycle counting method, this distribution is determined using specialized algorithms that, by analyzing the history of stress or strain in time, determine the distribution of amplitudes. Whereas in the case of the spectral method, certain parameters and coefficients are estimated, which are most often functions of the moments of the first five orders of the power spectral density function; and on the basis of these parameters, the amplitude distribution is approximated [6]. Due to the efficiency of numerical calculations, a significant increase in interest in the spectral method has been observed in recent years, which, in conjunction with the finite element method (FEM), allows determination of the fatigue life of machine elements and structures subjected to stochastic loading, taking into account the many aspects of structure dynamics. The spectral method is rarely used for notched elements or amplitudes above the yield stress, because, with the use of this classical approach we will obtain overestimated fatigue results. That is why it is necessary to propose a solution for the elastoplastic approach in order to estimate a fatigue life value that gets as close as possible to the real experimental value. Methods that use stress correction, due to stress values being over the yield point, are known, but are often shunned for fatigue calculations for a range above Hooke’s law. The algorithm for determining fatigue life in the case of the spectral method is usually limited to the use of statistical information for stress loading courses. However, it is rare to use deformation loading histories for calculations. Various studies have highlighted the influence of strain rate on structure, as well as fracture behavior [7,8,9]. Another limitation of the spectral method is the inability to take into account elastoplastic deformations, including the inability to include notched elements without special transformations in regards to the amplitudes. There have been some approaches to model plasticity; for example, by Rognon et al. [10], but they only tested in the case of equivalent von Mises strain power spectral density (PSD). The von Mises approach has been defined for frequency domain calculations by Pitoistet and Pneumont [11]. This approach is not always valid in terms of calculations because it has some incoherencies which may affect the PSD, as noted in the paper by Benasciutti [12]. The method discussed in this work presents the use of elastoplastic deformations in the calculation process of fatigue life, using the spectral method. For this purpose, 0H18N9 (EN 1.4301, DIN 304) steel tests for pendulum bending loads were carried out by the authors on their recently designed experimental test stand. The main goal was to verify the implementation of methods of correction of linear elastic stress to equivalent elastoplastic stress in the frequency domain. We reached it using the algorithm for calculating fatigue life in terms of the spectral method.

## 2. Materials and Methods

**Figure 1:**
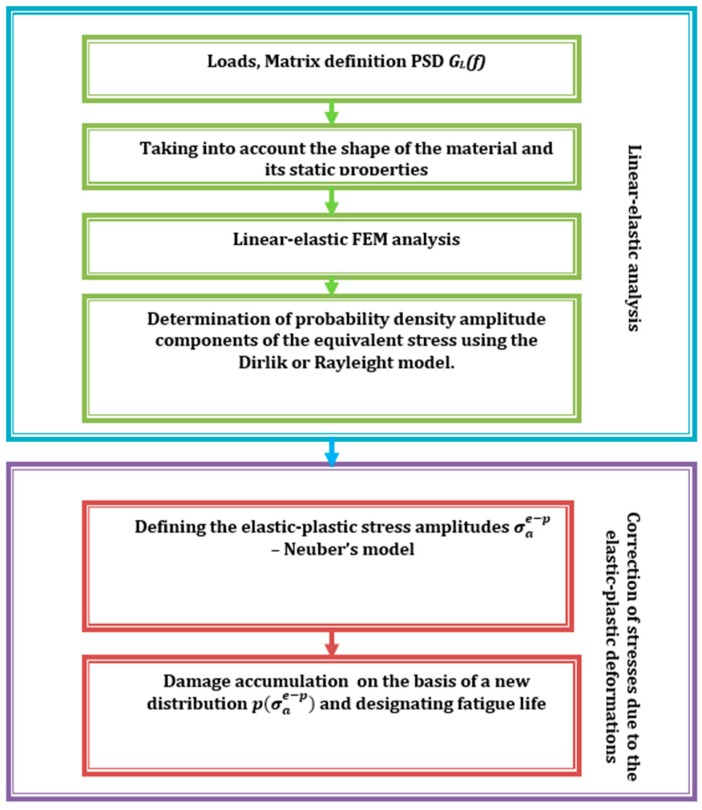
Calculation algorithm for determining fatigue life using the spectral method and stress correction due to elastoplastic deformation.

A solution was undertaken and a method presented, in which it is necessary to correct the stress course if the time history is unknown in the spectral method and only the function of the spectral density of the power of the stress course is known. This would be possible by adopting known methods of correction of linear elastic stresses, e.g., implementation in accordance with the Neuber hypothesis [13], and with the spectral method by performing corrections directly on the probability distribution of amplitudes estimated in the frequency domain. Popular algorithms for determining fatigue life consist of a series of blocks in which appropriate calculations or transformations are performed. An example algorithm is shown in Figure 1. Due to the complexity of the phenomenon of fatigue and the large number of factors affecting it, during the construction of models, a phenomenological description of this phenomenon was used, while striving to obtain an equivalent in the sense of a fatigue uniaxial loading state, e.g., tensile compression. Obtaining such a state is necessary to use the basic fatigue characteristics of a material and to determine durability in a subsequent calculation step.

Spectral methods are currently being developed especially in terms of calculations for non-Gaussian loads [14,15]. However, the problem of taking into account the impact of local elastoplastic deformations on computational fatigue life in the spectral method remains.

**Figure 2:**
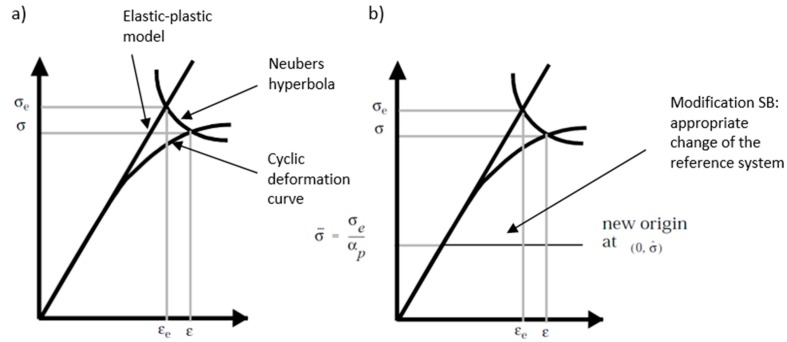
Mertens–Dittmann (**a**) and Seeger–Bestea (**b**) transformation based on [17].

Our approach was proposed in order to avoid the use of time history, which means that we concentrated on the use of the PSD of the stress course in the frequency domain. In order to reach our goal, we adopted known methods of correction of linear elastic stresses by performing corrections directly on the amplitude probability distribution estimated in the spectral method calculation using, e.g., Neuber correction using the appropriate transformation (Figure 2). During the determination of fatigue life with the use of the spectral method, the distribution of probability density *p(σ_a_)* is determined based on the spectral density of stress from the linear elastic analysis, which has to be used. This treatment gave good results for proportional loads and materials corresponding to the Masing rule [16] (the Masing material model is often used in solid mechanics to describe materials with stable elastoplastic properties). Using Masing’s rule in relation to fatigue tests, one can understand the special properties of a material, which are manifested stably under cyclic loads. Such a material, with the same load parameters (force or bending moment), draws the same path on a graph (*ε-σ*) for each subsequent load cycle. Hysteresis loops snap during a fatigue test under constant amplitude fatigue loading. Therefore, we needed to find corresponding stress amplitudes for the elastic and elastoplastic values, which corresponded to the Neuber hyperbola for the intersection points with the elastoplastic model and cyclic deformation curve. These new amplitudes were then used in the process of new probability density estimations with the correction for the elastoplastic stress values.

**Figure 3:**
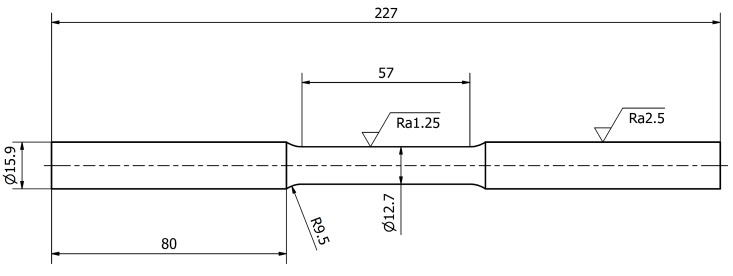
Shape and dimensions of the 0H18N9 tensile test specimen.

**Figure 4:**
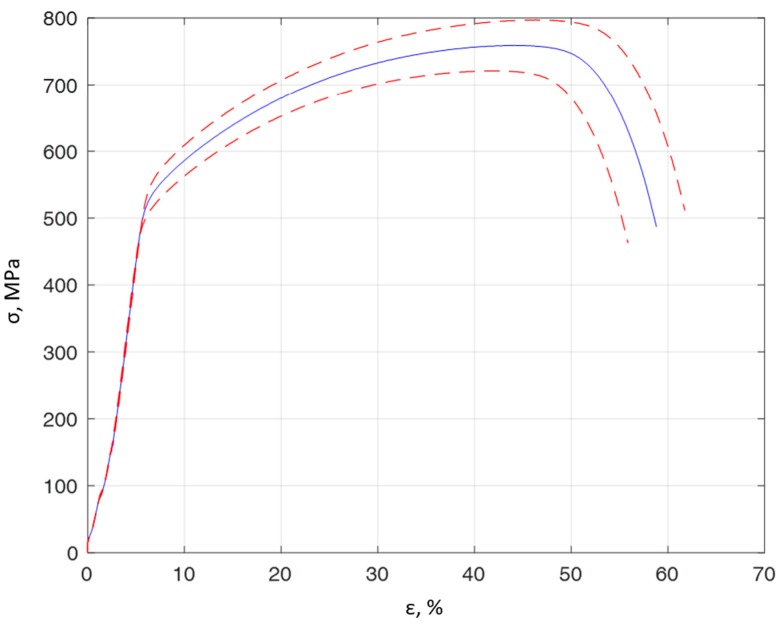
The average tensile curve for 0H18N9 steel for 6 specimens (solid line blue color) with a standard deviation of 5% (dashed lines red color).

The tests were carried out for stainless steel specimens of 0H18N9 (EN 1.4301, DIN 304). The first tests included a tensile test. For this purpose, specimens were prepared in accordance with ASTM standards and are presented in Figure 3. Basic mechanical properties, such as the ultimate strength *R_m_*, yield strength *R_e_*, Young modulus *E*, and Poisson ratio *υ*, are presented in Table 1 for the average of 6 specimens. A stretching diagram is shown in Figure 4.

**Figure 5:**
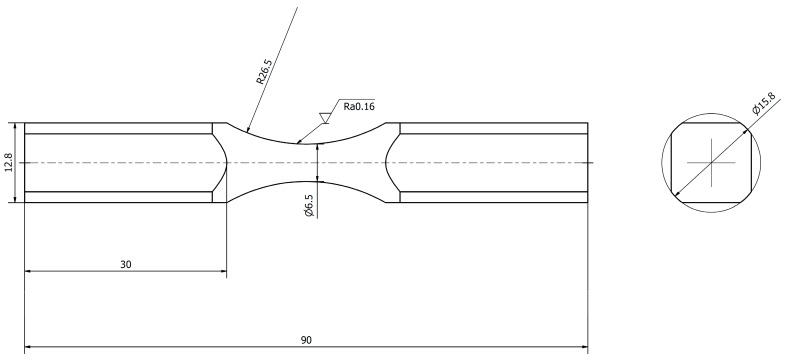
Shape and dimensions of a diabolo specimen made of 0H18N9 steel for fatigue tests.

Before physically preparing the specimens for fatigue tests, several versions of the specimen were prepared using different shapes and dimensions, which were then modeled using the finite element method in order to obtain information on the maximum deformations and stresses arising in the specimen. Figure 5 shows the shapes and dimensions of the specimens used during the tests.

**Figure 6:**
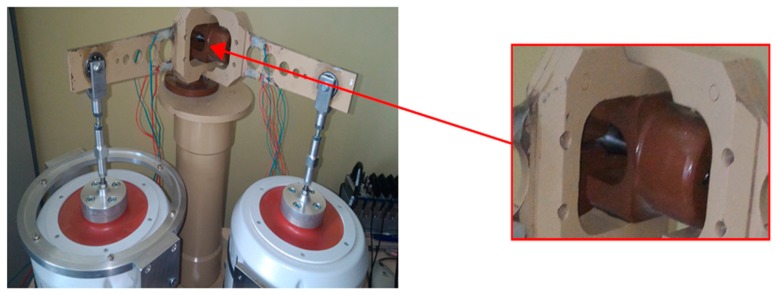
Test stand for tests with uniaxial and biaxial load. The image on the right shows a magnification of the position in which the specimen is clamped inside the stand.

Random fatigue tests were performed on an experimental designed test stand [18] for uniaxial and biaxial load conditions, able to test bending, torsion, and a combination of bending and torsion (Figure 6). Currently, it is possible to perform tests on a predefined power spectral density for each individual lever.

**Figure 7:**
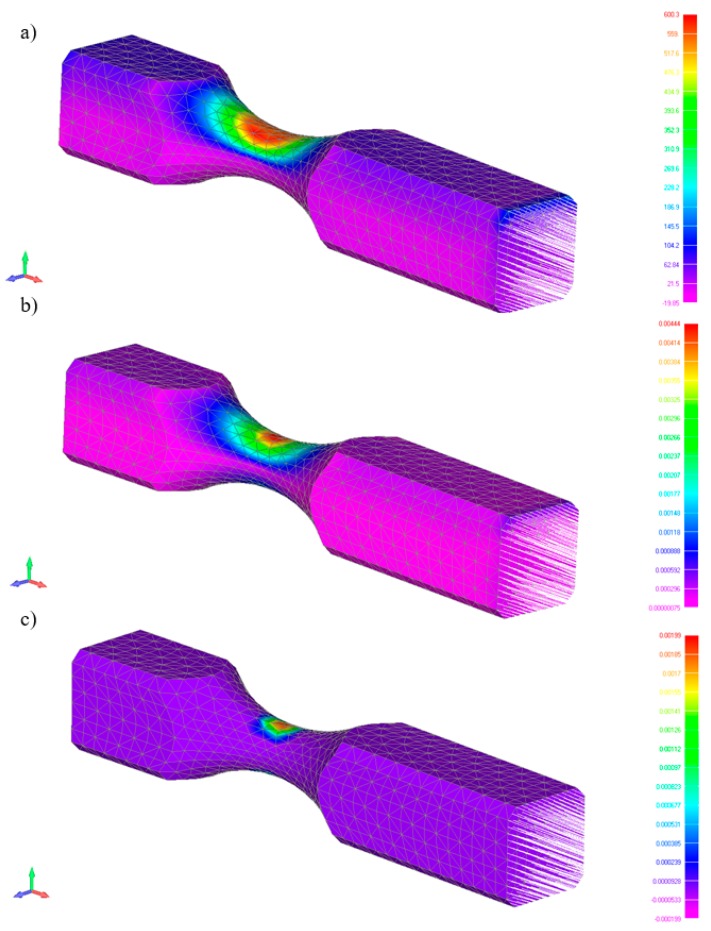
Calculations: Maximum stress (**a**), maximum deformation (**b**), and maximum plastic deformation for specimen (**c**).

The maximum specimen load used for FEM calculations was based on the maximum inductor strength for random loads and on the maximum inductor stroke. Calculations of maximum stresses and strains, including plastic zones, using information for the elastoplastic range, are presented in Figure 7.

**Figure 8:**
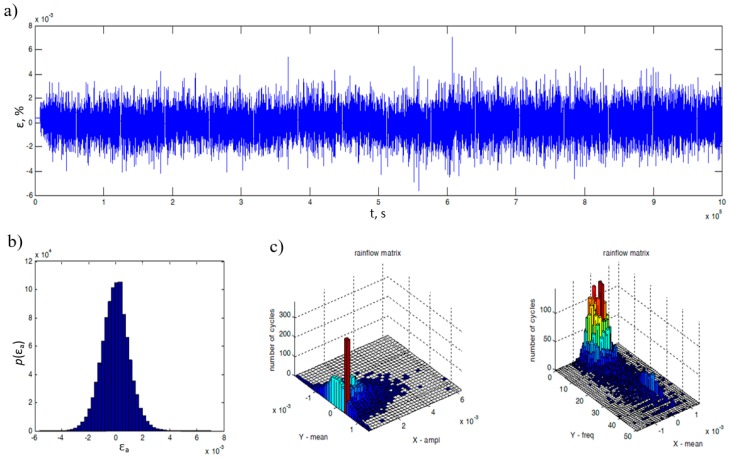
A fragment of the deformation process registered with the strain gauges (**a**), distribution of deformation amplitudes (**b**), and its characteristics due to matrices counted by the rainflow method (**c**).

The working principle of the experimental test stand can be explained with the use of the specimens used during the tests. Specimens with strain gauges glued to their surfaces are fixed in the handle, where both levers connect with the main column, as shown in Figure 6. Then, at the end of the lever a force is applied, which transmits the bending momentum to the specimen. The control is equipped with a random signal generator. The control system maintains the appropriate shape of the power spectral density. The specimens were subjected to a Gaussian narrow band loading signal. A section of one of the registered waveforms, and its statistics in the time domain, are shown in Figure 8. The statistical information that serve as important information are the rainflow amplitude distribution and the rainflow matrix of strain amplitudes.

**Figure 9:**
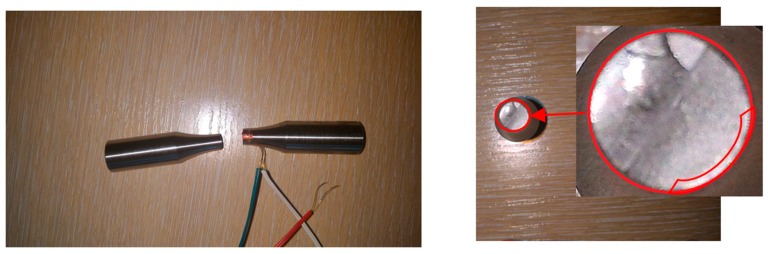
Scrap obtained during random bending tests with a crack initiation site.

The research was carried out until crack initiation was observed and then the registration process was stopped. Specimens were then damaged to obtain information about the nature of the crack and interesting scrap areas within the sample cross section (Figure 9).

The calculation procedure used was based on the stress course. To model the elastoplastic deformation, the constitutive model was needed to connect the current strain state to the accumulated plastic strain (damage to the material). The authors are aware of strain hardening (non-linear) deformation of material, but it was not included in the calculation procedure. Basic fatigue stress characteristics like the Basquin curve were also used [19]. Stress amplitudes $\sigma_{a}^{e}$ used in damage accumulation were corrected to bring their values closer to those obtained with elastoplastic models. The assumptions for this correction are energetic and used with the Ramberg–Osgood model [20,21]:

(1) $$\varepsilon_{a}{}^{e-p}\;=\;\frac{\sigma_{a}^{e-p}}{E}+{\left(\frac{\sigma_{a}^{e-p}}{\kappa }\right)}^{\frac{1}{n}}$$

where κ—cyclic deformation coefficient of reinforcement, *n*—cyclic strain hardening coefficient, *σ_a_*—elastic stress amplitude (superscript e) or elastoplastic stress (superscript e−p), and *E*—Young’s modulus.

**Figure 10:**
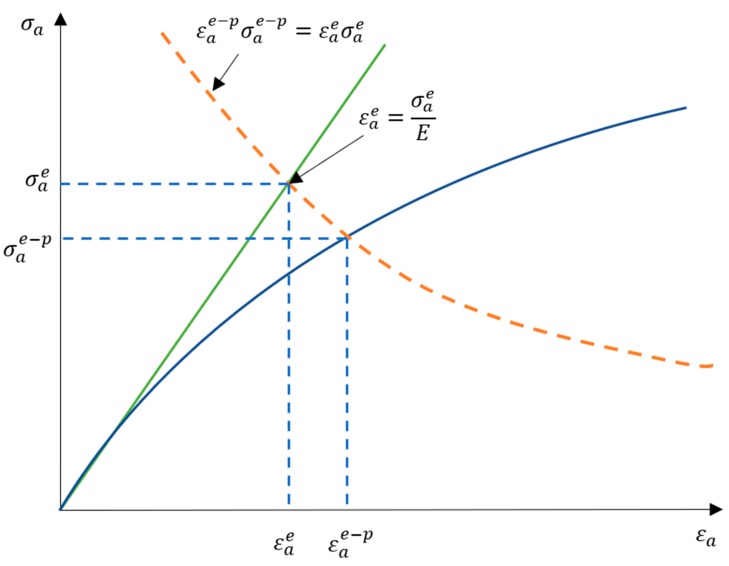
Neuber hyperbola together with the linear elastoplastic model (Hooke’s law) and deformation curve used in the derivation of the model.

If we draw a Neuber hyperbola on the elastoplastic linear model of the material and deformation curve, such as that in Figure 10, we can see that the hyperbola describes the relation:

(2) $$\varepsilon_{a}^{e-p}\sigma_{a}^{e-p}\;=\;\varepsilon_{a}^{e}\sigma_{a}^{e}$$

This means that we could formulate elastic strain amplitude $\varepsilon_{a}^{e}$ with the use of the elastic stress amplitude and Young modulus *E*:

(3) $$\varepsilon_{a}^{e-p}\sigma_{a}^{e-p}\;=\;\frac{\sigma_{a}^{e}}{E}\sigma_{a}^{e}$$

Based on the appropriate transformations of the above equation, we obtain:

(4) $$\varepsilon_{a}{}^{e-p}\;=\;\frac{{\left(\sigma_{a}^{e}\right)}^{2}}{E\sigma_{a}^{e-p}}$$

Then, we can use the obtained equation and substitute it into the Ramberg–Osgood model

(5) $$\frac{{\left(\sigma_{a}^{e}\right)}^{2}}{E\sigma_{a}^{e-p}}\;=\;\frac{\sigma_{a}^{e-p}}{E}+{\left(\frac{\sigma_{a}^{e-p}}{\kappa }\right)}^{\frac{1}{n}}$$

One of the most important features of such an algorithm is the correct correction of stresses determined by a linear elastic model for values corresponding to the stresses noted in typical construction materials. Modeled as an elastoplastic body we obtain:

(6) $$\sigma_{a}^{e}\;=\;\sqrt{{\left(\sigma_{a}^{e-p}\right)}^{2}+{\left(\frac{\sigma_{a}^{e-p}}{\kappa }\right)}^{\frac{1}{n}}E\sigma_{a}^{e-p}}$$

With the help of the last formula, stress amplitudes $\sigma_{a}^{e-p}$ can be numerically determined for the material described by the Ramberg–Osgood model [20], based on stress amplitudes $\sigma_{a}^{e}$ for a linear elastic material (FEM). This formula is used to create the corrected distribution in regards to the elastoplastic behavior.

## 3. Results

**Figure 11:**
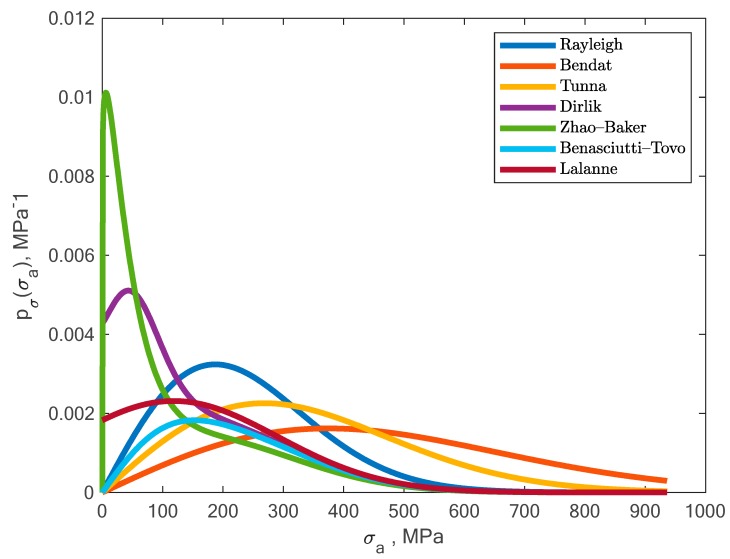
Obtained shapes of a wide range of probability distribution models after transformation due to elastoplastic deformations.

Calculations were performed according to the procedure presented in the algorithm (Figure 1). Stress amplitudes after correction due to elastoplastic deformations were used to determine the new stress amplitude distribution, which was then used in the process of determining durability. For this purpose, one can use one of the many distributions used to calculate durability. Figure 11 presents a graph with a wide range of probability distribution models after transformation due to elastoplastic deformations. For the purpose of further calculations, we chose four of the most popular probability density functions, and most of them are commonly used in commercial software.

Calculations were performed for these given probability density functions:

For the Dirlik model [22]:

(7) $$p\left(\sigma_{a}\right)\;=\;\frac{1}{2\sqrt{\xi_{0}}}\cdot \left[\frac{K_{1}}{K_{4}}\cdot e^{\frac{-Z}{K_{4}}}+\frac{K_{2}\cdot Z}{R^{2}}\cdot e^{\frac{-Z^{2}}{2\cdot R^{2}}}+K_{3}\cdot Ze^{\frac{-Z^{2}}{2}}\right]$$

where *ξ_i_* moments obtained from the power spectral densities for *i* = 0,…,4, *K*_1_, *K*_2_, *K*_3_, *K*_4_, and Z are model coefficients described in detail elsewhere [23,24].

For the Zhao–Baker model [25]:

(8) $$p\left(\sigma_{a}\right)\;=\;w\alpha \beta \sigma^{\left(\beta -1\right)}exp\left(-\alpha \sigma^{b}\right)+\left(1-w\right)\sigma \;exp\left(\frac{-\sigma^{2}}{2}\right)$$

where *σ*, *w, α, β* are factors described as [25]:

(9) $$\sigma \;=\;\frac{2\sigma_{a}}{\sqrt{\mu }}$$

(10) $$w\;=\;\frac{1-\gamma }{1-\sqrt{\frac{2}{\pi }}\Gamma \left(1+\frac{1}{\beta }\right)\alpha^{\frac{-1}{\beta }}}$$

(11) α = 8−7γ

(12) $$\beta \;=\;\{\begin{array}{cc} 1.1 & \gamma <0.9\\ 1.1+9\left(\gamma -0.9\right) & \gamma \geq 0.9 \end{array}$$

(13) $$\gamma \;=\;\frac{\xi_{2}}{\sqrt{\xi_{0}\xi_{4}}}$$

where *μ* is the variance.

For the Benasciutti–Tovo model [3]:

(14) $$p\left(\sigma_{a}\right)\;=\;b\frac{\gamma \sigma_{a}}{\xi_{0}}exp\left(\frac{-\sigma_{a}^{2}\gamma }{2}\right)+\left(1-b\right)\frac{\sigma_{a}}{\gamma^{2}\xi_{0}}exp\left(\frac{-\sigma_{a}^{2}\gamma^{2}\xi_{0}}{2}\right)$$

where b—weight function dependent from the PSD.

For the Lalanne model [26]:

(15) $$p\left(\sigma_{a}\right)\;=\;\sqrt{\frac{1-\gamma^{2}}{2\pi \xi_{0}}}exp\left(\frac{-\sigma_{a}{}^{2}}{2\xi_{0}\left(1-\gamma^{2}\right)}\right)+\frac{\gamma \sigma_{a}}{2\xi_{0}}exp\left(\frac{-\sigma_{a}{}^{2}}{2\xi_{0}}\right)\left(1-erf\left(\frac{\gamma \sigma_{a}}{\sqrt{2\xi_{0}\left(1-\gamma^{2}\right)}}\right)\right)$$

Finally, we substitute all required relationships into the following equation [27]:

(16) $$T_{cal}\;=\;\frac{1}{M^{+}\int_{0}^{\infty }\frac{p\left(\sigma_{a}\right)}{N_{f}\left(\sigma_{a}\right)}d\sigma_{a}}$$

where *M^+^*—is the expected number of peaks per unit of time, *N_f_*—is the number of cycles, and *p(σ_a_)*—stress amplitude probability distribution.

**Figure 12:**
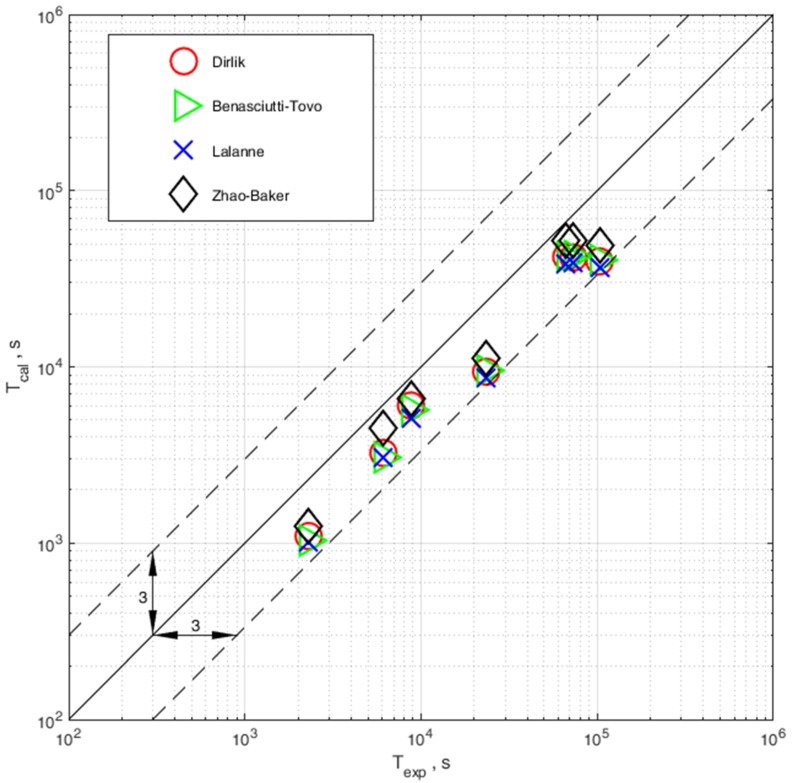
Comparison of experimental and computational durability calculated with the use of four probability density distributions (Dirlik, Benasciutti–Tovo, Lalanne and Zhao–Baker).

The results of the comparison of computational and experimental durability are shown in Figure 12. The comparison was made only for specimens with strain gauges stuck at the place of occurrence of the largest surface stresses, in accordance with the points identified using FEM.

## 4. Discussion

**Figure 13:**
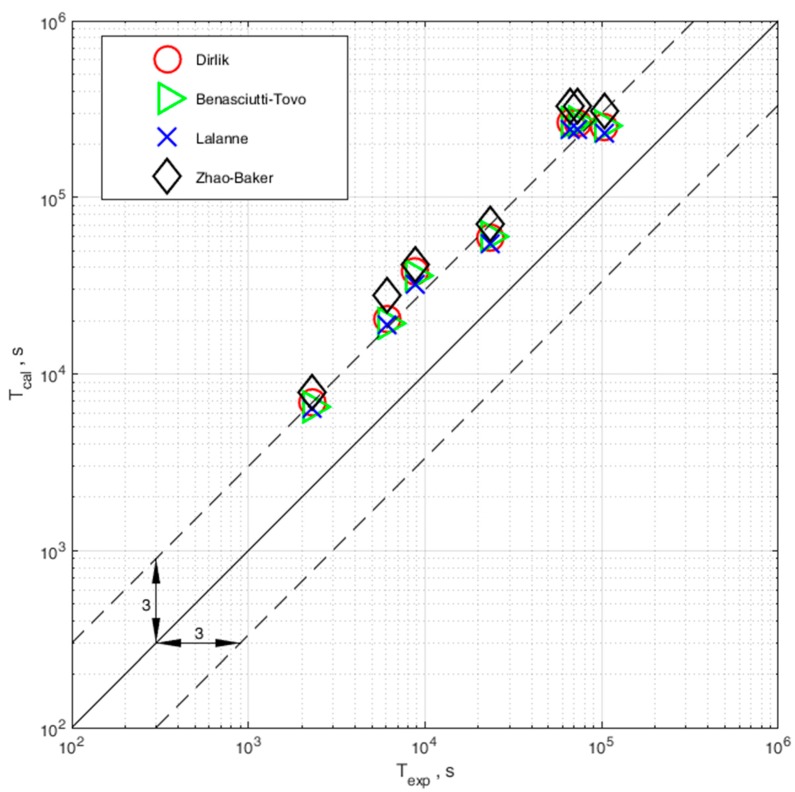
Comparison of experimental and computational durability calculated with the use of four probability density distributions (Dirlik, Benasciutti–Tovo, Lalanne, and Zhao–Baker), but no elastoplastic correction.

With elastoplastic correction, the fatigue life will automatically reduce and, clearly, the obtained calculation results do not overestimate the experimental data, which can be seen if we perform calculations without correction, as presented in Figure 13. All calculation results without correction are on the unsafe side of the comparison.

One of the most important elements of the presented algorithm is the appropriate correction of stresses determined by the linear elastic model for values corresponding to the stresses registered with the use of strain gauges for typical construction materials, modeled as an elastoplastic body. Corrections of this type are currently popular in fatigue calculations using the finite element method. It should be noted that they are used only for calculations of time-domain durability, i.e., for algorithms using the cycle counting procedure. In frequency defined methods, we do not have access to the time history, but we have the corresponding cycles for each stress level in the form of the distribution. These are later transformed in terms of their elastoplastic behaviors. The problem associated with the use of this correction has been solved for the frequency domain by the proposed algorithm, which results in the ability to use the spectral method for a range of stresses above the cyclic yield strength of a material.

We can, therefore, assume that it is possible to adapt the stress correction methods previously used in the time domain determined in accordance with the body’s linear elastic model to the frequency domain; as a result, the range of applicability of the spectral method for determining FEM assisted fatigue life will be extended to the range of the average number of cycles and machine components and notched structures. The main idea is to use the algorithm in the spectral method of determining fatigue life with elastoplastic stress correction being used when determining fatigue life, assisted by FEM calculations. This type of correction has not yet been implemented in frequency domain methods. Future plans will be to use this correction, among others, based on the hypotheses of Molski–Glinka [17], Łagoda–Macha [28], and others. It is important to note that the Neuber transformation is used to estimate stresses and deformations in the area of notches based on the stresses determined by linear elastic FEM analysis. This method is implemented in most commercial programs for determining fatigue life, e.g., in the MSC Fatigue program [29] there are two variants of this transformation: Mertens–Dittmann and Seeger–Bestea. The main idea of these transformations is to assume the equality of products of stress and nominal strain (subscript e) and local stress and strain (elastoplastic values):

(17) $$\varepsilon_{a}^{e}\sigma_{a}^{e}\;=\;\varepsilon_{a}^{e-p}\sigma_{a}^{e-p}$$

This allows you to plot the so-called Neuber hyperbola on a chart, as presented earlier in Figure 10. The intersection point of Neuber hyperbola with the cyclic strain diagram indicates the strain values and the elastoplastic stress. This should be the next step in future publications; to test these assumptions for the other variants of transformation.

## 5. Conclusions and Observations

The influence of the elastoplastic strain on the fatigue life estimation method defined in the frequency domain has been presented. The method has been verified with the use of experimental fatigue test results for 0H18N9 steel for the case on uniaxial bending loading state with a random probability distribution. The following conclusions and observations can be drawn:

- An algorithm for taking into account the elastoplastic strain in the process of fatigue life assessment with the use of spectral method is presented.
- In order to use the proposed correction, we need to use the Neuber hyperbola to obtain the values corresponding to the elastic stress amplitudes obtained during the probability density calculation and their corresponding elastoplastic stress amplitudes.
- Correction due to elastoplastic stress is successfully applied to determine fatigue life in combination with all four distribution models.
- In the case of no correction, in terms of elastoplastic strain, we obtain overestimated fatigue calculation results.
- Comparison of experimental and computational durability with correction shows that calculations are within a safe scatter band of 3.
- All models used to calculate the probability density function enable obtaining results in the desired scatter band, and the computation results are on the safe side, as they do not overestimate the experimental results.

## Figures and Tables

**Table 1:** Important mechanical properties of 0H18N9 steel.

*R_m_* MPa	*R_e_* MPa	*E* GPa	*υ*
750	515	200	0.29
